# Updates on Morphea: Role of Vascular Injury and Advances in Treatment

**DOI:** 10.1155/2013/467808

**Published:** 2013-11-12

**Authors:** Julio C. Sartori-Valinotti, Megha M. Tollefson, Ann M. Reed

**Affiliations:** ^1^The Department of Dermatology, Mayo Clinic College of Medicine, Rochester, MN 55905, USA; ^2^The Department of Pediatrics, Mayo Clinic College of Medicine, Rochester, MN 55905, USA

## Abstract

Morphea and systemic sclerosis are fibrosing disorders of the skin that share common inflammatory and immunologic pathways that are responsible for the vascular changes, increased collagen production, and extracellular matrix proliferation seen in both conditions. Recent advances in molecular biology techniques have furthered our knowledge of the potential underlying pathogenic mechanisms and offer new and provocative areas of research for novel diagnostic and therapeutic interventions. This review focuses on the role of vascular injury in the development of morphea, the use of ultrasonography as a diagnostic modality, and well-established and newly proposed treatments.

## 1. Introduction

Morphea is an inflammatory, fibrosing skin disorder that leads to sclerosis of the dermis and subcutaneous tissue but in some cases may also extend to the fascia, muscle, and underlying bone. Clinically, morphea has an asymmetric distribution and is usually confined to one body area; hence it is also referred to as localized scleroderma. Systemic sclerosis (SSc), however, in addition to symmetric skin changes is characterized by internal organ involvement, sclerodactyly, presence of Raynaud's phenomenon, and nailfold capillary abnormalities. Despite these differences, both entities share common inflammatory and immunologic pathways that are ultimately responsible for the vascular changes, increased collagen production, and extracellular matrix proliferation seen in both conditions. Although the etiology and precise mechanisms that trigger the cascade of molecular events that culminate in skin fibrosis are not fully understood, advances in molecular biology techniques have furthered our knowledge of the potential culprits and offer new and provocative areas of research for novel diagnostic and therapeutic interventions. This brief review will focus on the role of vascular injury in the development of morphea with emphasis on recent basic research data as well as use of ultrasonography as a diagnostic method. Lastly, well-established and newly proposed treatments will be discussed. 

## 2. Clinical Features

Several classification systems have been developed in attempt to grasp the breath of the various forms of presentation of morphea [1, 2]. They are largely based on clinical findings and include, with minor differences, at least four major variants: plaque-type, linear, generalized and a miscellaneous group of morphologically distinct phenotypes.

**Figure 1:**
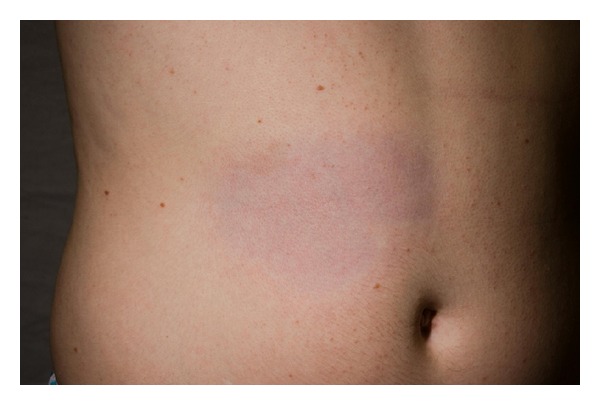
Plaque-type morphea.

*Plaque-Type Morphea (Morphea en Plaque or Circumscribed).* It is the most common subtype overall and the most common variant in adults. Most often located on the trunk, it begins as an erythematous-to-violaceous, edematous plaque of several centimeters that extends peripherally over a period of 3 to 5 years before it reaches a plateau phase. This is followed by an involution phase that leaves behind atrophic skin (Figure 1).

**Figure 2:**
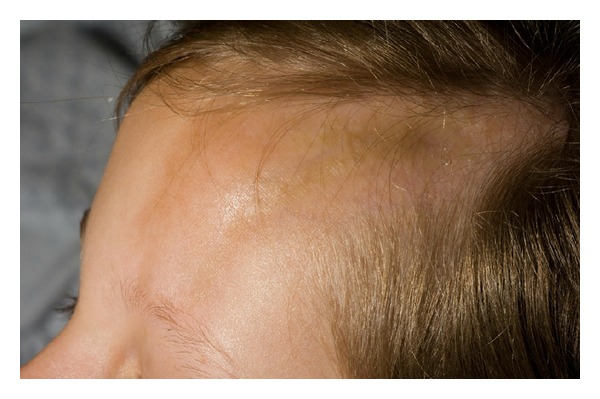
Morphea en coup de sabre.

**Figure 3:**
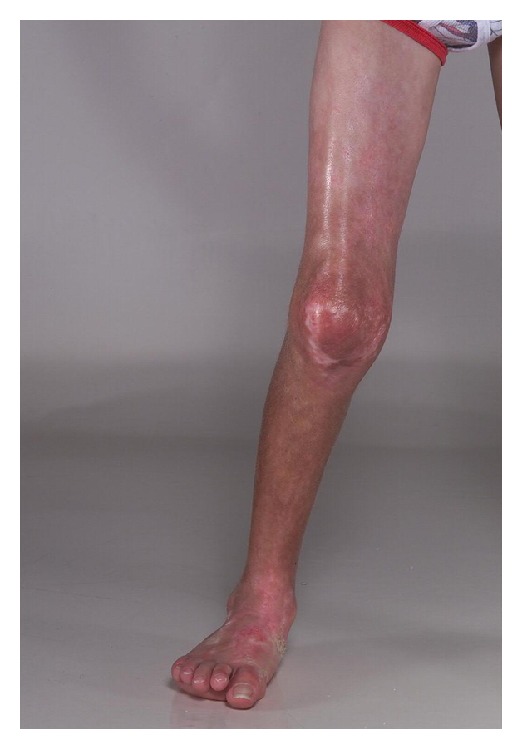
Linear morphea affecting the leg.

**Figure 4:**
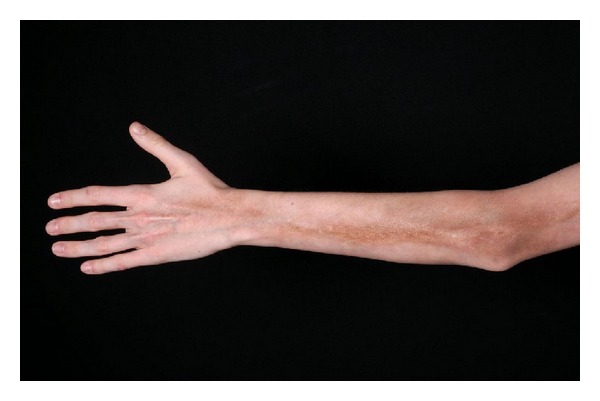
Linear morphea of the upper extremity.

**Figure 5:**
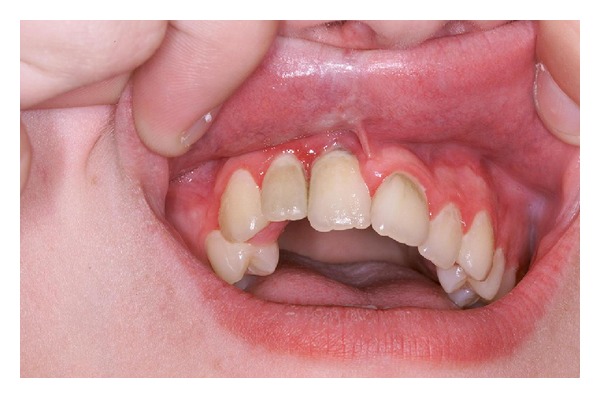
Linear morphea of the mouth. Note subtle changes of the inner upper lip and gingival mucosa around the maxillary right central incisor.

*Linear Morphea (Including Morphea en Coup de Sabre (Figure 2) and Progressive Hemifacial Atrophy or Parry-Romberg Syndrome).* Most common in children and adolescents, it presents as a linear induration on the scalp, forehead, trunk, or extremities (Figures 3 and 4), sometimes with involvement of the eye (in the case of facial lesions), underlying fascia, muscle, and bone. The latter may lead to limb atrophy and joint immobilization. Patients with Parry-Romberg syndrome and *en coup de sabre* morphea may also have seizures, headaches, and abnormal intracranial findings on magnetic resonance imaging (MRI) [3]. Linear morphea affecting the mouth has also been described (Figure 5). Antinuclear antibodies, ssDNA, and antihistones antibodies are usually positive.

*Generalized Morphea.* It is defined by the presence of four or more plaque-type lesions affecting two or more body sites or by the insidious onset of a slowly progressing plaque-type morphea on the trunk with eventual involvement of the entire trunk leading to progressive dyspnea due to mechanical restriction of chest cage expansion. Similar to linear morphea, patients in this subgroup have positive serology for antinuclear antibodies, ssDNA, and antihistones antibodies and are more likely to have constitutional symptoms.

*Miscellaneous Group.* Encompasses a variety of phenotypically different lesions including nodular, mixed (combination of two or more variants), guttate, bullous morphea and atrophoderma of Pasini and Pierini.

Irrespective of the clinical subgroup, morphea can be distinguished from SSc by the absence of internal organ involvement, Raynaud's phenomenon and nailfold capillary changes. On the other hand, SSc can be further subdivided into limited SSc (lSSc) and diffuse SSc (dSSC) on the basis of the extent and distribution pattern of skin disease. Sclerodermatous skin changes distal to the elbows or knees are referred to as lSSC whereas skin thickening proximal to these anatomic landmarks are characteristic of dSSC. A subset of patients with lSSc and calcinosis, Raynaud's phenomenon, esophageal dysmotility, sclerodactyly, and telangiectasias comprised the so-called CREST syndrome.

## 3. Pathogenesis

*Vascular Injury as the Crucial Event.* It has been proposed that endothelial cell damage may represent the initial and pivotal step in the development of soft tissue changes in morphea and systemic sclerosis. For example, using whole-field digital microscopy and transmission electron microscopy, Frech and coworkers demonstrated that 20 patients with SSc had increased skin interstitial edema, fibrosis, basal lamina lamellation, and endothelial swelling compared to normal controls, irrespective of disease duration, or appreciable clinical features [4]. This is consistent with the clinical findings of Raynaud's phenomenon and nail fold capillary changes seen in the early stages of the disease prior to the development of frank fibrosis. Infection, hypoxia, trauma, radiation, reactive oxygen species, and antiendothelial cell autoantibodies contribute to vascular injury and subsequent recruitment and activation of T and B lymphocytes and mononuclear cells, secretion of proinflammatory mediators and growth factors, endothelial cell apoptosis, and fibroblast activation which in turn leads to vascular and tissue remodeling and fibrosis [5–9].

Under physiologic and pathologic conditions, disruption of the capillary network results in decreased blood flow and tissue ischemia. The ability to withstand hypoxia varies by tissue type and is tightly regulated by hypoxia inducible factors. One of the adaptive responses to diminished tissue oxygen delivery is the formation of new vessels via either angiogenesis and/or vasculogenesis. The former refers to the formation of new vessels from preexisting vessels whereas vasculogenesis represents de novo vessel formation from bone marrow derived endothelial precursor cells (EPC). Cumulative evidence suggests that both processes are defective in SSc despite strong proangiogenic stimuli [10].

 Viruses may trigger vascular damage via neointimal proliferation and apoptosis likely through overproduction of profibrotic cytokines including TGF-beta, PDGF-alpha, and PDGF-beta [11]. Cytomegalovirus (CMV) RNA transcripts have been found in the endothelium of patients with sclerodermoid changes [12]. Similarly, parvovirus B19-infected endothelial cells, fibroblasts, and perivascular inflammatory cells of SSc patients have increased expression of TNF-alpha [13] which has been shown to participate in regulation of fibroblast function and endothelial activation [14]. A role for viral infection is further supported by the observation that molecular mimicry between human CMV late protein UL94 and NAG-2, a surface molecule present on endothelial cells and dermal fibroblasts, is responsible for cross-reactivity of human anti-CMV antibodies against the latter and may contribute to chronic sclerodermoid graft versus host disease (GVHD) [15, 16].

Endothelial cell apoptosis is a key feature of SSc and arguably the earliest event [17]. IL-6 and the Fas-pathway have been implicated in endothelial cell apoptosis [18, 19] via mechanisms dependent on the presence of neutrophils and antibody-induced cell-mediated toxicity, respectively. Circulating angiogenic cells are also prone to and undergo apoptosis in SSc through phagocytosis of microparticles and stimulation of acid sphingomyelinase activity [20]. Plasma samples of SSc patients have significantly higher levels of microparticles [21]. They are small, membrane-bound vesicles with altered surface lipids that participate in intercellular signaling [22]. Conversely, dermal fibroblasts are resistant to Fas-mediated apoptosis, perhaps due to deficiency in acid sphingomyelinase, and increased levels of anti-apoptotic proteins cFLIPs and cIAP, partially explaining their survival and contribution to increased extracellular matrix deposition in SSc [23, 24].

Antiendothelial cell autoantibodies (AECAs) likely promote vascular injury, endothelial cell apoptosis, generation of reactive oxygen species, and expression of adhesion molecules on endothelial cells in patients with SSc. They are a heterogeneous group of antibodies against endothelial cell-specific proteins and are present in 22–86% of patients with SSc [25]. Upon interaction with these antibodies, endothelial cells augment the expression of vascular cell adhesion molecule-1 (VCAM-1), intercellular adhesion molecule-1 (ICAM-1), and E-selectin resulting in increased leucocyte adhesion. Moreover, they stimulate platelet-derived growth factor (PDGF) pathway and oxidative stress [26]. Beyond their pathogenic role in dermal fibrosis, AECAs have also been linked to complications of SSs, including pulmonary fibrosis and hypertension, and apoptosis of bone marrow EPC [27, 28].

Microarray analyses of EPC from patients with SSc reveal a differential protein expression profile under both basal and hypoxic conditions, with differentiation towards a proinflammatory state. Furthermore, immunohistochemistry of SSc skin samples shows downregulation of TNFS10, TNFAIP3, and HOX-A9 and overexpression of PTGS-2 [29]. Most recently, genomic DNA analysis of eight pairs of monozygotic twins with SSc identified sites that were preferentially hypermethylated or hypomethylated on the X chromosome and corresponded to target genes governing, among other cellular pathways, apoptosis (MTM1), inflammation (ARAF), and oxidative stress (ENOX2) [30]. These latter findings provide some insight into the molecular alterations behind the higher prevalence of morphea and SSc in women. 

Vascular abnormalities seem not to be limited to the skin. Patients with SSc have reduced bone marrow vascularity, in spite of normal cell morphology, as measured by microvessel density. Notoriously, peripheral blood mononuclear cells (PMBC) from individuals with SSc release greater amounts of VEGF [31], and its expression is much higher in SSc patients [32, 33] thus suggesting a diminished responsiveness to angiogenic stimuli. Impaired production of TNF-like weak inducer of apoptosis (TWEAK), a newly characterized cytokine, by PMBC may be accountable for aberrant angiogenesis and tissue remodeling in SSc [34]. Lastly, fibroblasts and autoimmunity are also important pathogenic players in morphea and SSc, but in-depth discussion of these topics is beyond the scope of this review.

## 4. Evaluation

Based on the increasing evidence that vascular changes dominate the early stages of disease development, it is not surprising that there is emerging research looking into ways to rapidly and accurately recognize them. Ultrasonography has gained particular attention as a noninvasive, harmless, and inexpensive diagnostic tool. 

Morphea and SSc are characterized by three distinct phases of skin disease: active or edematous; inactive, sclerotic, or fibrosis; and atrophic lesions. Early recognition of the active phase may have both therapeutic and prognostic implications. In a study of 104 morphea lesions, ultrasonography was not inferior to dermatopathologic examination in evaluating active disease. Indeed, when compared to histology, increased cutaneous (dermal or subcutaneous) blood flow and hyperechogenicity of the subcutaneous tissue had both 100% specificity and 100% sensitivity [35]. In keeping with these observations, using fourteen MHz ultrasonography of 16 morphea lesions, hyperechogenicity correlated with the presence of moderate or severe sclerosis on histology. More importantly, ultrasonographic findings were more reliable than clinical-based scores such as the Modified Rodnan Skin Score (mRSS) [36] and have acceptable and reproducible inter- and intraobserver reliability. The inactive and atrophic phases of the disease also exhibit unique sonographic features [37, 38]. 

Ultrasound can additionally be used to determine the severity of musculoskeletal involvement [39] and endothelial function [40] in SSc and in sclerodermoid GVHD [41]. It has also been shown to be useful in monitoring the response to treatment. For instance, in pediatric patients, the hyperemia and increased echogenicity of active lesions disappeared after successful treatment [42]. In a different series, dermal thickness as measured by ultrasound was decreased in patients treated with phototherapy [43] and topical imiquimod [44]. Due to its depth of penetration, which is a function of frequency, the usefulness of ultrasonography is somewhat limited to the skin and subcutaneous tissue. In this regard MRI is more advantageous and allows for better assessment of deeper structures such as the fascia and underlying muscle [45, 46]. As with ultrasonography, MRI can be resourceful in monitoring disease activity and response to treatment [47].

## 5. Treatment

The treatment of morphea and skin disease in SSc is challenging, and its efficacy is difficult to assess owing to the absence of validated and standardized outcome measures. Nonetheless, numerous treatment modalities both systemic and topical have been investigated, the majority of which have been abandoned due to lack of response or have not been investigated in larger populations. However, among these interventions, methotrexate (MTX) alone or in combination with systemic steroids and phototherapy have been proven to be beneficial with stronger evidence to support their use.

### 5.1. Methotrexate

The effectiveness of methotrexate, primarily in conjunction with systemic steroids, has been validated by several retrospective studies. In the recent past, at least six prospective, including double-blind, randomized trials have confirmed the efficacy and safety of this therapeutic regimen. For example, in patients with juvenile morphea, clinical remission for a mean duration of 25 months was achieved with simultaneous use of MTX and prednisone [48]. In another study of pediatric patients with moderate to severe morphea, this combination strategy quickly resulted in clinical improvement, as determined by Modified LS Skin Severity Index, within two months of treatment [49]. Improvement in musculoskeletal involvement has also been observed in a prospective study of adults with deep morphea (mean age 52 years) [47]. When added to MTX and prednisone, imatinib, which inhibits fibroblast activity, halted the progression of skin disease and joint deformity in a 3-year-old patient [50]. MTX likely exerts its antifibrotic effects via inhibition of inflammatory cytokines such as IL-2, IL-4, IL-6, IL-8, and TNF-alpha and adhesion molecules such as ICAM-1 [7, 51, 52].

With regard to the use of MTX in SSc, in 2009 the European League Against Rheumatism/EULAR Scleroderma Trails and Research published recommendations for the management of the multiple manifestations of SSc, including cutaneous involvement. Based on two randomized controlled trials on patients with early diffuse SSc or limited SSc [53, 54], methotrexate was recommended as a first-line treatment for early diffuse SSc (Class A recommendation). Notwithstanding, MTX was superior to placebo in one of these studies [54], whereas the other showed only a trend favoring MTX, but it did not reach statistical significance. Two important considerations can be drawn from these conflicting observations. First, it is conceivable to hypothesize that, relative to morphea, the modest or lack of response to treatment with MTX in SSc is due to the fact that most studies for management of morphea included a combination of MTX and steroids. Second, with widespread involvement, the efficacy of MTX may be reduced or difficult to quantify. Placebo controlled trials assessing the benefits of combined MTX and systemic steroids for diffuse SSc are lacking.

 Mycophenolate mofetil is reserved as a second line agent that could be used for treatment of localized and generalized morphea after failed response to MTX and/or phototherapy [55, 56]. Over the past decade, B-cell depletion therapy has gained special attention as a successful intervention for various immune-mediated diseases. Pertaining to its use for sclerodermoid conditions, there are conflicting results in patients with refractory sclerodermoid GVHD either showing improvement [17] or lack of response [57]. A recent case report showed resolution of localized scleroderma with rituximab [58]. Larger case series and prospective studies will help elucidate its potential use as a standard treatment.

### 5.2. Phototherapy

First documented in 1994 [59], phototherapy for treatment of morphea has since been widely used and studied. By virtue of their longer wavelength and thus deeper penetration, PUVA therapy and UVA1 are the cornerstone of light treatment for localized scleroderma. Its mechanism of action likely involves the combination of various effects such as alteration in cytokine and growth factors expression, modulation of endothelial dysfunction, induction of matrix metalloproteinases that degrade collagen, apoptosis of Langerhans cells and T cells, and inhibition of collagen synthesis [60–63]. The treatment course varies among clinical protocols, but it typically requires approximately 30 sessions before clinical, histological, and ultrasonographic improvement can be appreciated. Furthermore, clinical improvement continues beyond cessation of therapy; thus prolonged treatment is neither needed nor indicated. Phototherapy is effective in all Fitzpatrick skin prototypes and is generally well tolerated, with no serious side effects. The main caveats to the use of UVA1 are the need for prolonged exposure times, diminished effectives after repetitive treatment owing to increased pigmentation, and its availability at specialized centers only. Alternatively, narrowband UVB and broadband UVA can be used with satisfactory results [64–67].

There is a paucity of data on the use of phototherapy for management of diffuse skin involvement in SSc, but PUVA and UVA1 have been reported to be effective. In a study of 18 patients with acrosclerosis, low dose UVA1 resulted in reduction of clinical score from 19.4 to 14.9; this was accompanied by elevation of dermal collagenase [68]. In a larger series of patients with different skin conditions amenable to treatment with UVA1, 12 patients with SSc/CREST had a moderate response (51–75% improvement) as determined by clinical assessment by the same physician before and after treatment [69]. Another study involving 3 patients with systemic scleroderma also reported improvement in the mRSS after UVA1 treatment [70]. Due to the small number of study subjects, evaluation of limited disease with involvement of the hands only, and the subjective (clinical) assessment of response to treatment, the results of these studies cannot be generalized. 

On the other hand, provocative data from the basic research literature may be key in providing the foundation for the development of new therapeutic interventions for morphea and SSc. For instance, the tight-skin (Tsk (−/+)) model of SSc shows abnormal fibrillin-1 expression and chronic oxidative damage that may be responsible for impaired angiogenesis [71]. Circulating endothelial cells and EPCs from patients with SSc treated with iloprost, a synthetic analogue of the vasodilatory prostacyclin PGI2, exhibit upregulation of antiapoptotic genes and genes involved in wound healing [72]. Treatment with recombinant human erythropoietin resulted in resolution of a nonhealing digital ulcer and reduction in apoptotic rates of bone marrow endothelial cells [73] in a patient with SSc. In conclusion, the advent of new technology has furthered our understanding of the imbricated mechanisms behind the development of these debilitating and disfiguring conditions. Nonetheless, placebo-controlled trials exploring these newly discovered pathways are much needed to expand our treatment repertoire. This task is rather challenging because, by virtue of its heterogenous presentation, better measures of disease activity and outcomes are necessary to accurately evaluate evidence-based therapies. Fortunately, research in this area is underway.

## References

[1] Laxer RM, Zulian F (2006). Localized scleroderma. *Current Opinion in Rheumatology*.

[2] Peterson LS, Nelson AM, Su WPD (1995). Classification of morphea (localized scleroderma). *Mayo Clinic Proceedings*.

[3] Chiu YE, Vora S, Kwon EK, Maheshwari M (2012). A significant proportion of children with morphea en coup de sabre and Parry-Romberg syndrome have neuroimaging findings. *Pediatric Dermatology*.

[4] Frech TM, Revelo MP, Drakos SG (2012). Vascular leak is a central feature in the pathogenesis of systemic sclerosis. *The Journal of Rheumatology*.

[5] Hasegawa M, Sato S, Nagaoka T, Fujimoto M, Takehara K (2003). Serum levels of tumor necrosis factor and interleukin-13 are elevated in patients with localized scleroderma. *Dermatology*.

[6] Higley H, Persichitte K, Chu S, Waegell W, Vancheeswaran R, Black C (1994). Immunocytochemical localization and serologic detection of transforming growth factor *β*1: association with type I procollagen and inflammatory cell markers in diffuse and limited systemic sclerosis, morphea, and Raynaud’s phenomenon. *Arthritis and Rheumatism*.

[7] Ihn H, Sato S, Fujimoto M, Kikuchi K, Takehara K (1995). Demonstration of interleukin-2, interleukin-4 and interleukin-6 in sera from patients with localized scleroderma. *Archives of Dermatological Research*.

[8] Kahari V-M, Sandberg M, Kalimo H, Vuorio T, Vuorio E (1988). Identification of fibroblasts responsible for increased collagen production in localized scleroderma by in situ hybridization. *Journal of Investigative Dermatology*.

[9] Sgonc R, Gruschwitz MS, Dietrich H, Recheis H, Gershwin ME, Wick G (1996). Endothelial cell apoptosis is a primary pathogenetic event underlying skin lesions in avian and human scleroderma. *The Journal of Clinical Investigation*.

[10] Cipriani P, Marrelli A, Liakouli V, Di Benedetto P, Giacomelli R (2011). Cellular players in angiogenesis during the course of systemic sclerosis. *Autoimmunity Reviews*.

[11] Hamamdzic D, Harley RA, Hazen-Martin D, LeRoy EC (2001). MCMV induces neointima in IFN-*γ*R-/- mice: intimal cell apoptosis and persistent proliferation of myofibroblasts. *BMC Musculoskeletal Disorders*.

[12] Magro CM, Crowson AN, Ferri C (2007). Cytomegalovirus-associated cutaneous vasculopathy and scleroderma sans inclusion body change. *Human Pathology*.

[13] Magro CM, Nuovo G, Ferri C, Crowson AN, Giuggioli D, Sebastiani M (2004). Parvoviral infection of endothelial cells and stromal fibroblasts: a possible pathogenetic role in scleroderma. *Journal of Cutaneous Pathology*.

[14] Koca SS, Isik A, Ozercan IH, Ustundag B, Evren B, Metin K (2008). Effectiveness of etanercept in bleomycin-induced experimental scleroderma. *Rheumatology*.

[15] Lunardi C, Dolcino M, Peterlana D (2006). Antibodies against human cytomegalovirus in the pathogenesis of systemic sclerosis: a gene array approach. *PLoS Medicine*.

[16] Pastano R, Dell'Agnola C, Bason C (2012). Antibodies against human cytomegalovirus late protein UL94 in the pathogenesis of scleroderma-like skin lesions in chronic graft-versus-host disease. *International Immunology*.

[17] Ratanatharathorn V, Ayash L, Reynolds C (2003). Treatment of chronic graft-versus-host disease with anti-CD20 chimeric monoclonal antibody. *Biology of Blood and Marrow Transplantation*.

[18] Barnes TC, Spiller DG, Anderson ME, Edwards SW, Moots RJ (2011). Endothelial activation and apoptosis mediated by neutrophil-dependent interleukin 6 trans-signalling: a novel target for systemic sclerosis?. *Annals of the Rheumatic Diseases*.

[19] Sgonc R, Gruschwitz MS, Boeck G, Sepp N, Gruber J, Wick G (2000). Endothelial cell apoptosis in systemic sclerosis is induced by antibody-dependent cell-mediated cytotoxicity via CD95. *Arthritis and Rheumatism*.

[20] Distler JHW, Akhmetshina A, Dees C (2011). Induction of apoptosis in circulating angiogenic cells by microparticles. *Arthritis and Rheumatism*.

[21] Guiducci S, Distler JHW, Jüngel A (2008). The relationship between plasma microparticles and disease manifestations in patients with systemic sclerosis. *Arthritis and Rheumatism*.

[22] Distler JHW, Huber LC, Gay S, Distler O, Pisetsky DS (2006). Microparticles as mediators of cellular cross-talk in inflammatory disease. *Autoimmunity*.

[23] Chabaud S, Corriveau M-P, Grodzicky T (2011). Decreased secretion of MMP by non-lesional late-stage scleroderma fibroblasts after selection via activation of the apoptotic fas-pathway. *Journal of Cellular Physiology*.

[24] Samuel GH, Lenna S, Bujor AM, Lafyatis R, Trojanowska M (2012). Acid sphingomyelinase deficiency contributes to resistance of scleroderma fibroblasts to Fas-mediated apoptosis. *Journal of Dermatological Science*.

[25] Mihai C, Tervaert JWC (2010). Anti-endothelial cell antibodies in systemic sclerosis. *Annals of the Rheumatic Diseases*.

[26] Abraham D, Distler O (2007). How does endothelial cell injury start? The role of endothelin in systemic sclerosis. *Arthritis Research and Therapy*.

[27] Dib H, Tamby MC, Bussone G (2012). Targets of anti-endothelial cell antibodies in pulmonary hypertension and scleroderma. *European Respiratory Journal*.

[28] Lewandowska K, Ciurzynski M, Gorska E (2013). Antiendothelial cells antibodies in patients with systemic sclerosis in relation to pulmonary hypertension and lung fibrosis. *Advances in Experimental Medicine and Biology*.

[29] Avouac J, Cagnard N, Distler JH (2011). Insights into the pathogenesis of systemic sclerosis based on the gene expression profile of progenitor-derived endothelial cells. *Arthritis and Rheumatism*.

[30] Selmi C, Feghali-Bostwick CA, Lleo A (2012). X chromosome gene methylation in peripheral lymphocytes from monozygotic twins discordant for scleroderma. *Clinical & Experimental Immunology*.

[31] Bielecki M, Kowal K, Lapinska A, Chwiesko-Minarowska S, Chyczewski L, Kowal-Bielecka O (2011). Peripheral blood mononuclear cells from patients with systemic sclerosis spontaneously secrete increased amounts of vascular endothelial growth factor (VEGF) already in the early stage of the disease. *Advances in Medical Sciences*.

[32] Carrai V, Miniati I, Guiducci S (2012). Evidence for reduced angiogenesis in bone marrow in SSc: immunohistochemistry and multiparametric computerized imaging analysis. *Rheumatology*.

[33] Del Papa N, Quirici N, Scavullo C (2010). Antiendothelial cell antibodies induce apoptosis of bone marrow endothelial progenitors in systemic sclerosis. *The Journal of Rheumatology*.

[34] Bielecki M, Kowal K, Lapinska A (2009). Diminished production of TWEAK by the peripheral blood mononuclear cells is associated with vascular involvement in patients with systemic sclerosis. *Folia Histochemica et Cytobiologica*.

[35] Wortsman X, Wortsman J, Sazunic I, Carreño L (2011). Activity assessment in morphea using color Doppler ultrasound. *Journal of the American Academy of Dermatology*.

[36] Nezafati KA, Cayce RL, Susa JS (2011). 14-MHz ultrasonography as an outcome measure in morphea (localized scleroderma). *Archives of Dermatology*.

[37] Li SC, Liebling MS, Haines KA, Weiss JE, Prann A (2011). Initial evaluation of an ultrasound measure for assessing the activity of skin lesions in juvenile localized scleroderma. *Arthritis Care and Research*.

[38] Szymańska E, Maj M, Majsterek M, Litniewski J, Nowicki A, Rudnicka L (2011). The usefulness of high frequency ultrasonography in dermatologlcal practice—ultrasound features of selected cutaneous lesions. *Polski Merkuriusz Lekarski*.

[39] Iagnocco A, Ceccarelli F, Vavala C (2012). Ultrasound in the assessment of musculoskeletal involvement in systemic sclerosis. *Medical Ultrasonography*.

[40] Fernandes TM, Bica BE, Villela NR (2012). Evaluation of endothelial function in patients with limited systemic sclerosis by use of brachial artery Doppler ultrasound. *Revista Brasileira de Reumatologia*.

[41] Osmola-Mankowska A, Silny W, Danczak-Pazdrowska A (2013). Assessment of chronic sclerodermoid Graft-versus-Host Disease patients, using 20 MHz high-frequency ultrasonography and cutometer methods. *Skin Research and Technology*.

[42] Li SC, Liebling MS, Haines KA (2007). Ultrasonography is a sensitive tool for monitoring localized scleroderma. *Rheumatology*.

[43] Buense R, Duarte IA, Bouer M (2012). Localized scleroderma: assessment of the therapeutic response to phototherapy. *Anais Brasileiros de Dermatologia*.

[44] Pope E, Doria AS, Theriault M, Mohanta A, Laxer RM (2011). Topical imiquimod 5% cream for pediatric plaque morphea: a prospective, multiple-baseline, open-label pilot study. *Dermatology*.

[45] Schanz S, Fierlbeck G, Ulmer A (2011). Localized scleroderma: MR findings and clinical features. *Radiology*.

[46] Schanz S, Henes J, Ulmer A (2013). Magnetic resonance imaging findings in patients with systemic scleroderma and musculoskeletal symptoms. *European Radiology*.

[47] Schanz S, Henes J, Ulmer A (2013). Response evaluation of musculoskeletal involvement in patients with deep morphea treated with methotrexate and prednisolone: a combined MRI and clinical approach. *American Journal of Roentgenology*.

[48] Zulian F, Vallongo C, Patrizi A (2012). A long-term follow-up study of methotrexate in juvenile localized scleroderma (morphea). *Journal of the American Academy of Dermatology*.

[49] Torok KS, Arkachaisri T (2012). Methotrexate and corticosteroids in the treatment of localized scleroderma: a standardized prospective longitudinal single-center study. *The Journal of Rheumatology*.

[50] Inamo Y, Ochiai T (2012). Successful combination treatment of a patient with progressive Juvenile Localized Scleroderma (Morphea) using Imatinib, Corticosteroids, and Methotrexate. *Pediatric Dermatology*.

[51] Reitamo S, Remitz A, Varga J (1993). Demonstration of interleukin 8 and autoantibodies to interleukin 8 in the serum of patients with systemic sclerosis and related disorders. *Archives of Dermatology*.

[52] Visvanathan S, Marini JC, Smolen JS (2007). Changes in biomarkers of inflammation and bone turnover and associations with clinical efficacy following infliximab plus methotrexate therapy in patients with early rheumatoid arthritis. *The Journal of Rheumatology*.

[53] Pope JE, Bellamy N, Seibold JR (2001). A randomized, controlled trial of methotrexate versus placebo in early diffuse scleroderma. *Arthritis and Rheumatism*.

[54] van den Hoogen FHJ, Boerbooms AMT, Swaak AJG, Rasker JJ, van Lier HJJ, van de Putte LBA (1996). Comparison of methotrexate with placebo in the treatment of systemic sclerosis: a 24 week randomized double-blind trial, followed by a 24 week observational trial. *British Journal of Rheumatology*.

[55] Derk CT, Grace E, Shenin M, Naik M, Schulz S, Xiong W (2009). A prospective open-label study of mycophenolate mofetil for the treatment of diffuse systemic sclerosis. *Rheumatology*.

[56] Fett N, Werth VP (2011). Update on morphea: part II. Outcome measures and treatment. *Journal of the American Academy of Dermatology*.

[57] George L, George B, Gottlieb DJ, Hertzberg M, Fernandez-Peñas P (2012). Lack of efficacy of rituximab in refractory sclerodermatous chronic GVHD. *Bone Marrow Transplantation*.

[58] Chimenti MS, Teoli M, Stefani AD, Giunta A, Esposito M, Perricone R (2013). Resolution with rituximab of localized scleroderma occurring during etanercept treatment in a patient with rheumatoid arthritis. *European Journal of Dermatology*.

[59] Kerscher M, Vokenandt M, Meurer M, Lehmann P, Plewig G, Rocken M (1994). Treatment of localised scleroderma with PUVA bath photochemotherapy. *The Lancet*.

[60] El-Mofty M, Mostafa W, Esmat S (2004). Suggested mechanisms of action of UVA phototherapy in morphea: a molecular study. *Photodermatology Photoimmunology and Photomedicine*.

[61] Morita A, Kobayashi K, Isomura I, Tsuji T, Krutmann J (2000). Ultraviolet A1 (340-400 nm) phototherapy for scleroderma in systemic sclerosis. *Journal of the American Academy of Dermatology*.

[62] Scharffetter K, Wlaschek M, Hogg A (1991). UVA irradiation induces collagenase in human dermal fibroblasts in vitro and in vivo. *Archives of Dermatological Research*.

[63] Stein B, Rahmsdorf HJ, Steffen A, Litfin M, Herrlich P (1989). UV-induced DNA damage is an intermediate step in UV-induced expression of human immunodeficiency virus type 1, collagenase, c-fos, and metallothionein. *Molecular and Cellular Biology*.

[64] El-Mofty M, Mostafa W, El-Darouty M (2004). Different low doses of broad-band UVA in the treatment of morphea and systemic sclerosis. A clinico-pathologic study. *Photodermatology Photoimmunology and Photomedicine*.

[65] El-Mofty M, Zaher H, Bosseila M, Yousef R, Saad B (2000). Low-dose broad-band UVA in morphea using a new method for evaluation. *Photodermatology Photoimmunology and Photomedicine*.

[66] Kreuter A, Hyun J, Stücker M, Sommer A, Altmeyer P, Gambichler T (2006). A randomized controlled study of low-dose UVA1, medium-dose UVA1, and narrowband UVB phototherapy in the treatment of localized scleroderma. *Journal of the American Academy of Dermatology*.

[67] Zwischenberger BA, Jacobe HT (2011). A systematic review of morphea treatments and therapeutic algorithm. *Journal of the American Academy of Dermatology*.

[68] Kreuter A, Breuckmann F, Uhle A (2004). Low-dose UVA1 phototherapy in systemic sclerosis: effects on acrosclerosis. *Journal of the American Academy of Dermatology*.

[69] Tuchinda C, Kerr HA, Taylor CR (2006). UVA1 phototherapy for cutaneous diseases: an experience of 92 cases in the United States. *Photodermatology Photoimmunology and Photomedicine*.

[70] Pereira N, Santiago F, Oliveira H, Figueiredo A (2012). Low-dose UVA1 phototherapy for scleroderma: what benefit can we expect?. *Journal of the European Academy of Dermatology and Venereology*.

[71] Xu H, Zaidi M, Struve J (2011). Abnormal fibrillin-1 expression and chronic oxidative stress mediate endothelial mesenchymal transition in a murine model of systemic sclerosis. *American Journal of Physiology—Cell Physiology*.

[72] Tinazzi E, Dolcino M, Puccetti A (2010). Gene expression profiling in circulating endothelial cells from systemic sclerosis patients shows an altered control of apoptosis and angiogenesis that is modified by iloprost infusion. *Arthritis Research and Therapy*.

[73] Ferri C, Giuggioli D, Manfredi A (2010). Recombinant human erythropoietin stimulates vasculogenesis and wound healing in a patient with systemic sclerosis complicated by severe skin ulcers. *Clinical and Experimental Dermatology*.

